# Supplementary material for: Structural basis for HIV-1 capsid adaption to a deficiency in IP6 packaging
Source: Nat Commun. 2025 Sep 1;16:8152. doi: 10.1038/s41467-025-63363-9 (PMC12402280; doi:10.1038/s41467-025-63363-9)
Supplement: Supplementary file 1 — Supplementary information [file 41467_2025_63363_MOESM1_ESM.pdf]

Supplementary figures and tables

Supplementary Table 1. CryoET data collection and structure determination of immature Gag

**Data acquisition**						
samples	K227A	KAKA/T8I	G225R	KAKA /G225R	KAKA/T8I /G225R	A77V/KAKA /T8I/G225R
Microscope	Titan Krios					
Voltage (keV)	300					
Detector	Gatan Quantum K3		Facon4i			
Energy-filter	Yes					
Slit width (eV)	20		10			
Super-resolution mode	No					
Å/pixel	1.34	1.34	1.501	1.501	1.501	1.501
Defocus range (µm)	-1.5 to -6					
Defocus increment (µm)	0.3					
Acquisition scheme	Dose-Symmetric, -60/60, 3° step, group 3					
Total Dose (electrons/Å^2)	123					
Number of Frames	10					
Number of Tomograms	71	81	78	86	61	89
**Structure determination**						
No. of subtomograms	141,486	159,659	284,966	165,561	116,132	228,256
Resolution (Å)	3.82	3.67	3.79	4.10	4.05	3.78
b-factor applied	-10	-10	-10	-10	-10	-10
Data deposition	EMD-51826	EMD-51825	EMD-51821	EMD-51822	EMD-51823	EMD-51824

Supplementary Table 2. Mutants deficient for IP6 packaging acquire mutations throughout CA upon propagation in T cell lines. The listed IP6-packaging-deficient mutants were transfected into the indicated T cells and propoagated. Virus from the peak of replication was collected and re-passaged in fresh cells. Sanger sequencing of genomic DNA isolated from infected cells was used to identify mutations acquired during propagation. .

Transfected Clone	Passages	Cells	Mutations
KTKA/M4L	2	C8166	N21S, A105T, T210S
KTKA/T8I	2	C8166	M68I
KAKA/T8I	1	C8166	M68I, T107N, G208R
KAKA/M4L	1	C8166	G208R, S146N
KTKA/T8I	1	C8166	M68I, H87Q, G208R
KTKA/M4L	1	C8166	G208R, Q192K, P207S
KAKA	2	MT4	G225S
KTKA	2	MT4	E187Q, P207T
KAKA/T8I	2	MT4	T216I
KTKA/T8I	2	MT4	G61E
KAKA/M4L/T8I	5	MT4	M68I, G94D
KAKA/M4L/T8I	2	MT4	A31D,G61E
KTKA/T8I/G225S	3	MT4	G61E
KAKA/T8I/G225S	3	MT4	T58I
KAKA/T216I/G225S	3	MT4	T58I
KAKA/T8I	3	MT4	T58I,M68I
KTKA/T8I	3	MT4	
KAKA	4	MT4	V11I, SP1-M4I
KTKA/T216I	4	MT4	SP1-M4I
KTKA/T8I/G225S	4	MT4	S225R
KTKA/T216I	2	C8166	H12Y, D156N, G225S
KTKA/T8I/G225S	2	C8166	M68I, G61E, S146N
KAKA/G225S	2	C8166	MA E40K
KAKA/T8I/G225S	2	C8166	A22T, P207S
KAKA/T8I/G225R	3	MT4	A77V,A88E

Supplementary Table 3. CryoEM data collection and structure determination of assembled mature capsid.

**Data collection and processing**			
KAKA/G225R CA	Hexamer	Tri-hexamer Classs 1	Tri-hexamer Classs 2
Microscope	Tian Krios		
Magnification	64,000		
Voltage	300 kV		
Electron dose	40 e-/Å^2		
Detector	Gatan Quantum K3		
Defocus Range	-0.8 to -2.5 µm		
Pixel Size	1.34 Å		
Symmetry	C6	C3	C3
Particle numbers	1,646,193	1,320,752	309,508
Map resolution(Å)	2.75	3.59	4.24
FSC threshold	0.143	0.143	0.143
Local resolution range(Å)	2.3-4.0	3.2-6.0	3.7-6.0
**Refinement**			
Initial Model used (PDB code)	AlphaFold3 Prediction		
Model resolution(Å)	3.5		
FSC threshold	0.5		
Map sharpening *B* factor(Å^2)	-130.3		
Model composition			
Non-hydrogen atoms	10374		
Residues	1338		
R.m.s.d deviations			
Bond length(Å)	0.003		
Bond angles(°)	0.941		
Validation			
MolProbity score	2.24		
Clashscore	12.16		
Rotamers Outliers (%)	2.23		
Ramachandran Plot			
Favored(%)	94.49		
Allowed(%)	5.51		
Outliers(%)	0.00		
Data deposition	EMD-52724<br>PDB:9I8I	EMD-52725	EMD-52726

Supplementary Table 4. Highest C-terminal segment (residues 220-231) contact occupancies at the CA dimer interface for KAKA/G225R CA, KAKA CA and WT CA from 400ns MD simulations.

KAKA/G225R CA		KAKA CA		WT CA	
**Residue interaction**	**Occupancy range (%)**	**Residue interaction**	**Occupancy range (%)**	**Residue interaction**	**Occupancy range (%)**
R225-D152	2 – 16	G225-D152	0 - 7	K227-D152	0 – 16
R229-N193	3 – 57	R229-D152	2 – 4	R229-N193	0 – 2
R229-Q192	3 – 57	L231-R154	1 – 4	R229-Q192	0 – 3
R229-D152	9 – 46	L231-K199	1 – 9	R229-D152	0 – 12
L231-R154	17 – 50			R229-D197	0 – 9
L231-K199	3 - 27			L231-Q192	0 – 16
				L231-R154	0 – 11
				L231-K199	0 - 7

Supplementary Table 5. Summary of CA systems built for molecular dynamics simulations.

System	Simulation description	Dimensions (Å)	No. atoms	No. water molecules	Salt concentration (mM NaCl)	Simulation length (ns)	No. replicas
KAKA/G225R CA trimer of dimers	NPT equilibration/production	172.5 x 174.0 x 88.4	267,858	81,896	150	400	7
KAKA CA trimer of dimers	NPT equilibration/production	172.5 x 174.0 x 88.4	267,750	81,896	150	400	7
WT CA trimer of dimers	NPT equilibration/production	172.5 x 174.0 x 88.5	267,894	81,896	150	400	7

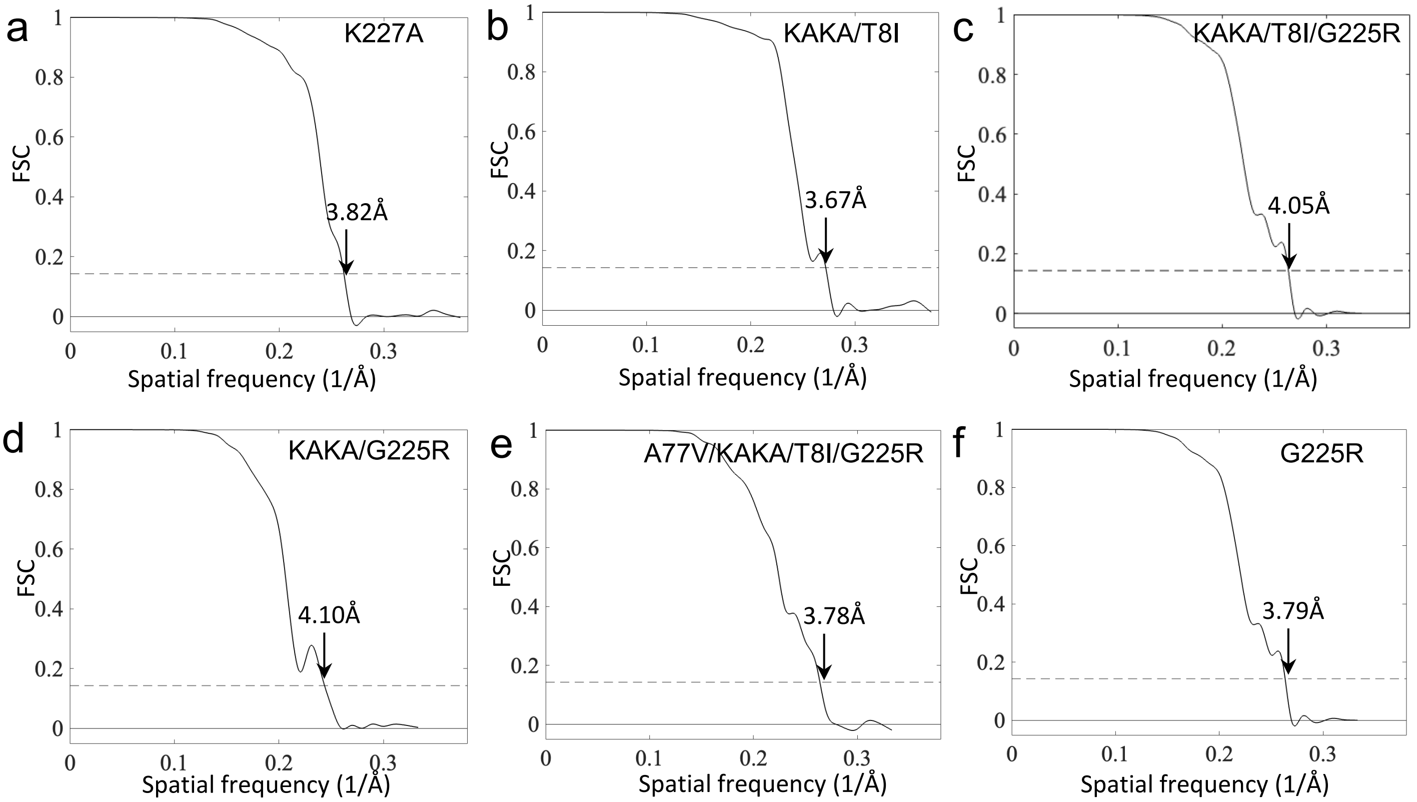

Supplementary Figure 1 | Fourier Shell Correlation (FSC) plot of immature Gag density maps. (a-f) Fourier Shell Correlation (FSC) plot of cryoET STA density maps from K227A, KAKA/T8I, KAKA/T8I/G225R, KAKA/G225R, A77V/KAKA/T8I/G225R and G225R VLPs. The resolution is indicated at the FSC value of 0.143.

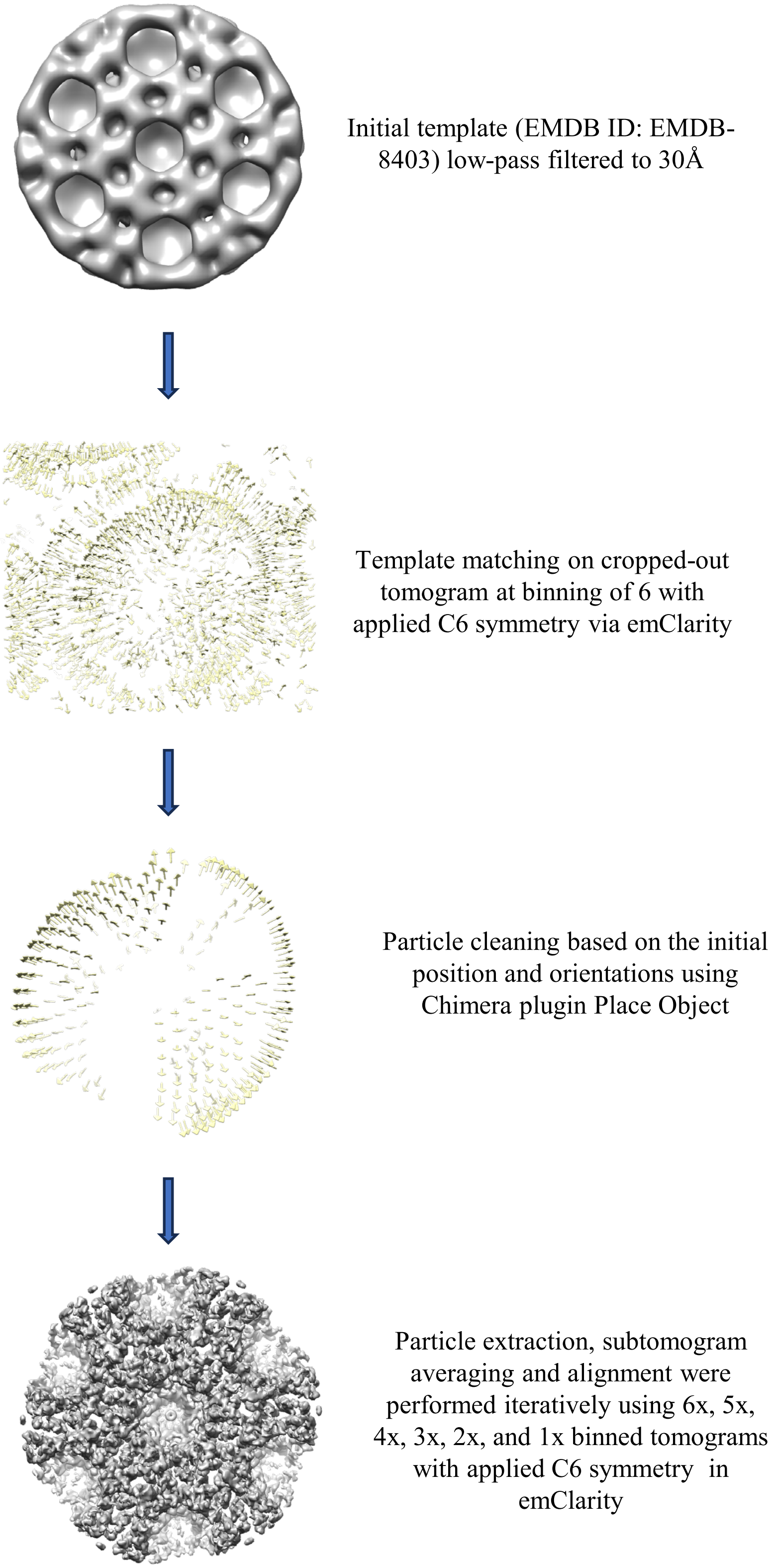

Supplementary Figure 2 | The workflow of croyET STA data processing in emClarity

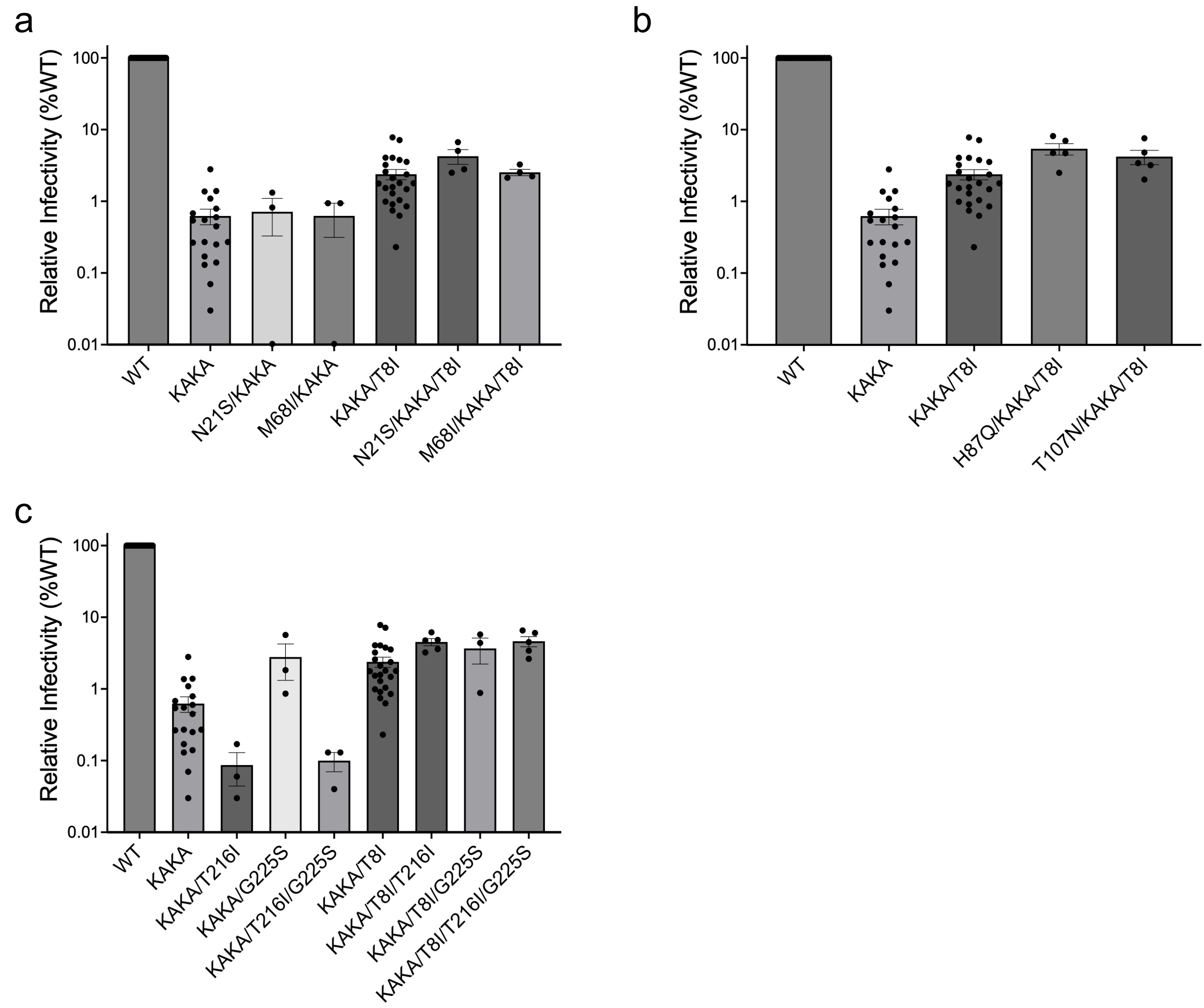

Supplementary Figure 3 | Infectivity of compensatory mutants in combination with KAKA and KAKA/T8I. (a-c) Mutations acquired during propagation of IP6-packaging-deficient mutants were introduced into KAKA and KAKA/T8I pNL4-3 vectors and infectivity in TZM-bl cells was measured at 36-48 hours post-infection and expressed as a percentage of WT. Data are the mean of at least three independent biological replicates for each mutant and error bars depict +/- SEM.Precise n for each group: (a) WT=24, KAKA=19, KAKA/T8I=24, N21S/KAKA=3, M68I/KAKA=3, KAKA/T8I=24, N21S/KAKA/T8I=4, M68I/KAKA/T8I=4 (b) WT=24, KAKA=19, KAKA/T8I=24, H87Q/KAKA/T8I=5, T107N/KAKA/T8I=5 (c) WT=24, KAKA=19, KAKA/T216I=3, KAKA/G225S=3, KAKA/T216I/G225S=3, KAKA/T8I=24, KAKA/T8I/T216I=5, KAKA/T8I/G225S=3, KAKA/T8I/T216I/G225S=5. Infectivity means for WT, KAKA, and KAKA/T8I are the same presented in a-c and in Figure 1i. Source data are provided as a Source Data file.

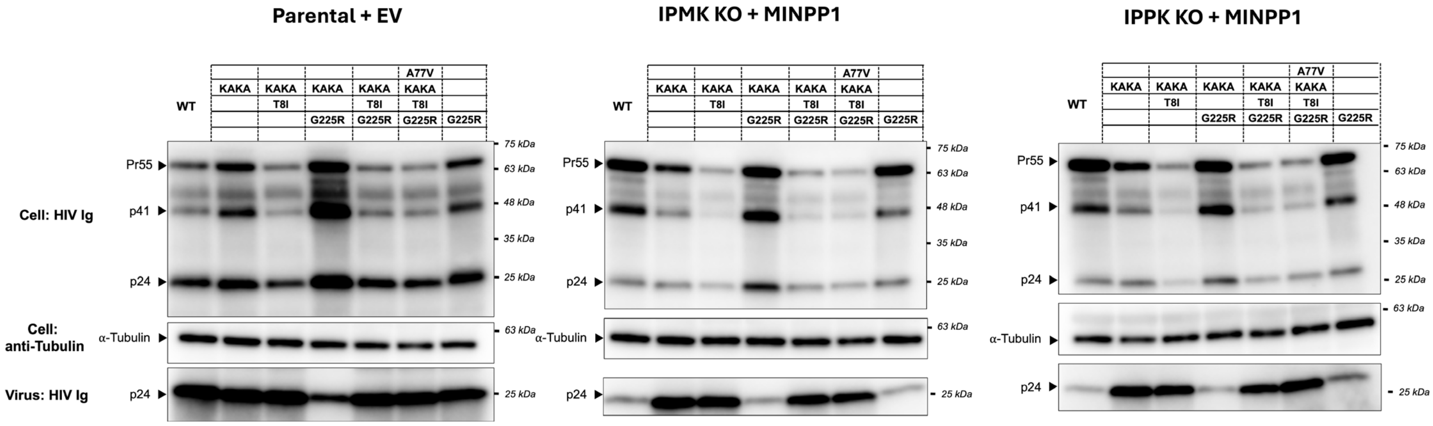

Supplementary Figure 4 | Western blot analysis for virus production efficiency measurements. Representative western blots are shown for cell and viral lysates collected from 293T Parental cells co-transfected with pNL4-3 and empty vector (EV) (left), and 293T IPMK KO (middle) and IPPK KO cells co-transfected with pNL4-3 and a MINPP1 expression vector. Cellular Gag and virus p24 were quantified, and virus production efficiency was calculated in each cell line as described in the Materials and Methods. Four independent experiments were performed with similar results. Cell and virus lysates were run on separate gels for each experiment and were processed in parallel. Source data are provided as a Source Data file.

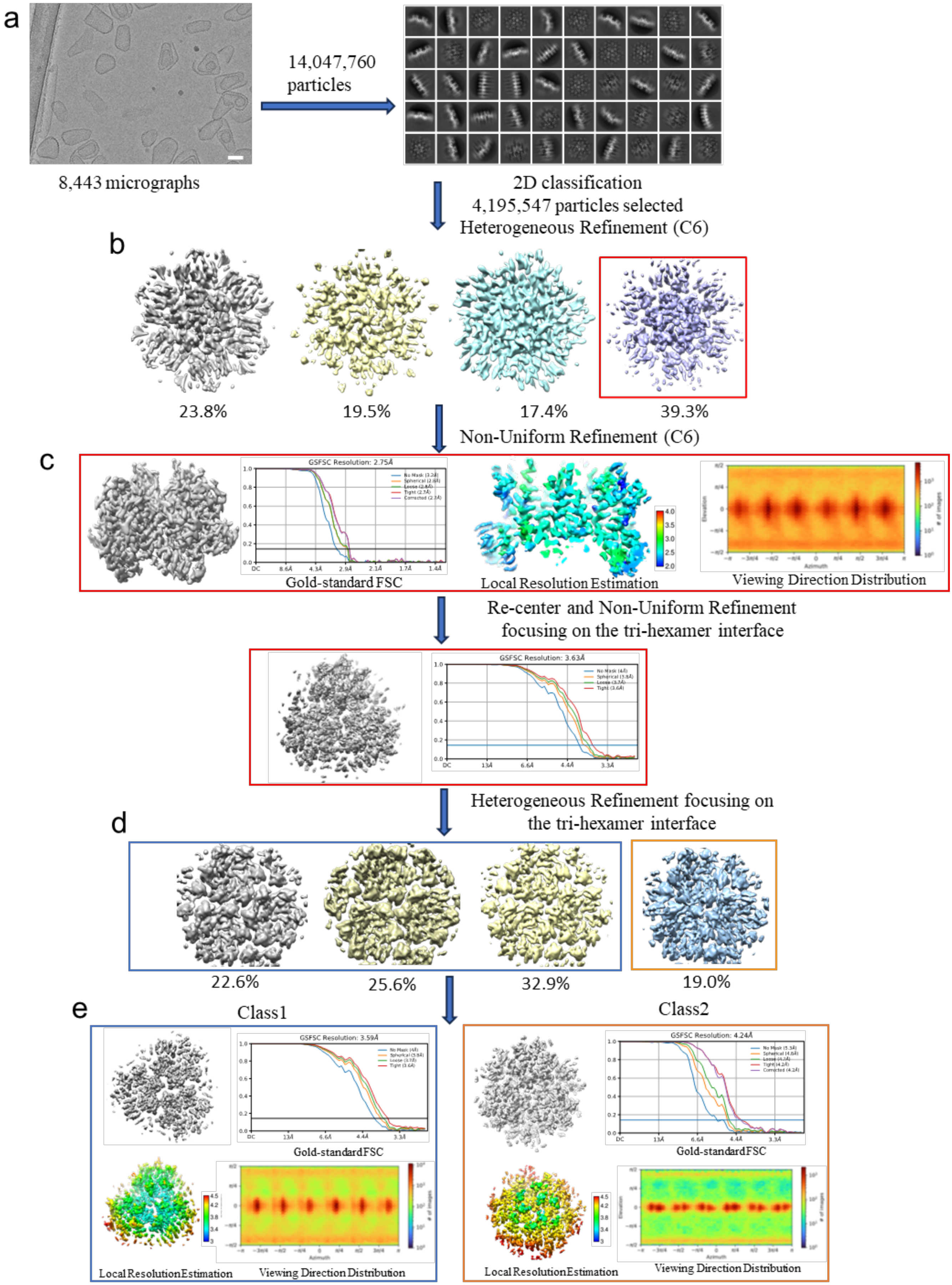

a
14,047,760
particles
8,443 micrographs
2D classification
4,195,547 particles selected
Heterogeneous Refinement (C6)
b
23.8%
19.5%
17.4%
39.3%
Non-Uniform Refinement (C6)
c
GSFSC Resolution: 2.75Å
Gold-standard FSC
Local Resolution Estimation
Viewing Direction Distribution
Re-center and Non-Uniform Refinement
focusing on the tri-hexamer interface
GSFSC Resolution: 3.63Å
Heterogeneous Refinement focusing on
the tri-hexamer interface
d
22.6%
25.6%
32.9%
19.0%
e
Class1
Class2
GSFSC Resolution: 3.59Å
Gold-standard FSC
GSFSC Resolution: 4.24Å
Gold-standard FSC
Local Resolution Estimation
Viewing Direction Distribution
Local Resolution Estimation
Viewing Direction Distribution

Supplementary Figure 5 | CryoEM structural determination of KAKA/G225R CA capsid hexamer. (a) Step1: Reference-free particle picking and 2d class averaging to remove bad particles. (b)Step2: Heterogeneous refinement with C6 symmetry applied. (c) Step3: Non-uniform refinement with the best class selected for Step2. Gold standard FSC and local resolution estimation with 0.143 cutoff, and orientation distribution for cryoSPARC are shown on the right. (d) Step4: Re-center the particles on the tri-hexamer interface and perform heterogenous refinement focusing on the tri-hexamer interface. (e) Step5: Subgroup to two classes based on the density of CA-C terminus tail and perform Non-uniform refinement respectively. Gold standard FSC and local resolution estimation with 0.143 cutoff, and orientation distribution for cryoSPARC are shown for each class.

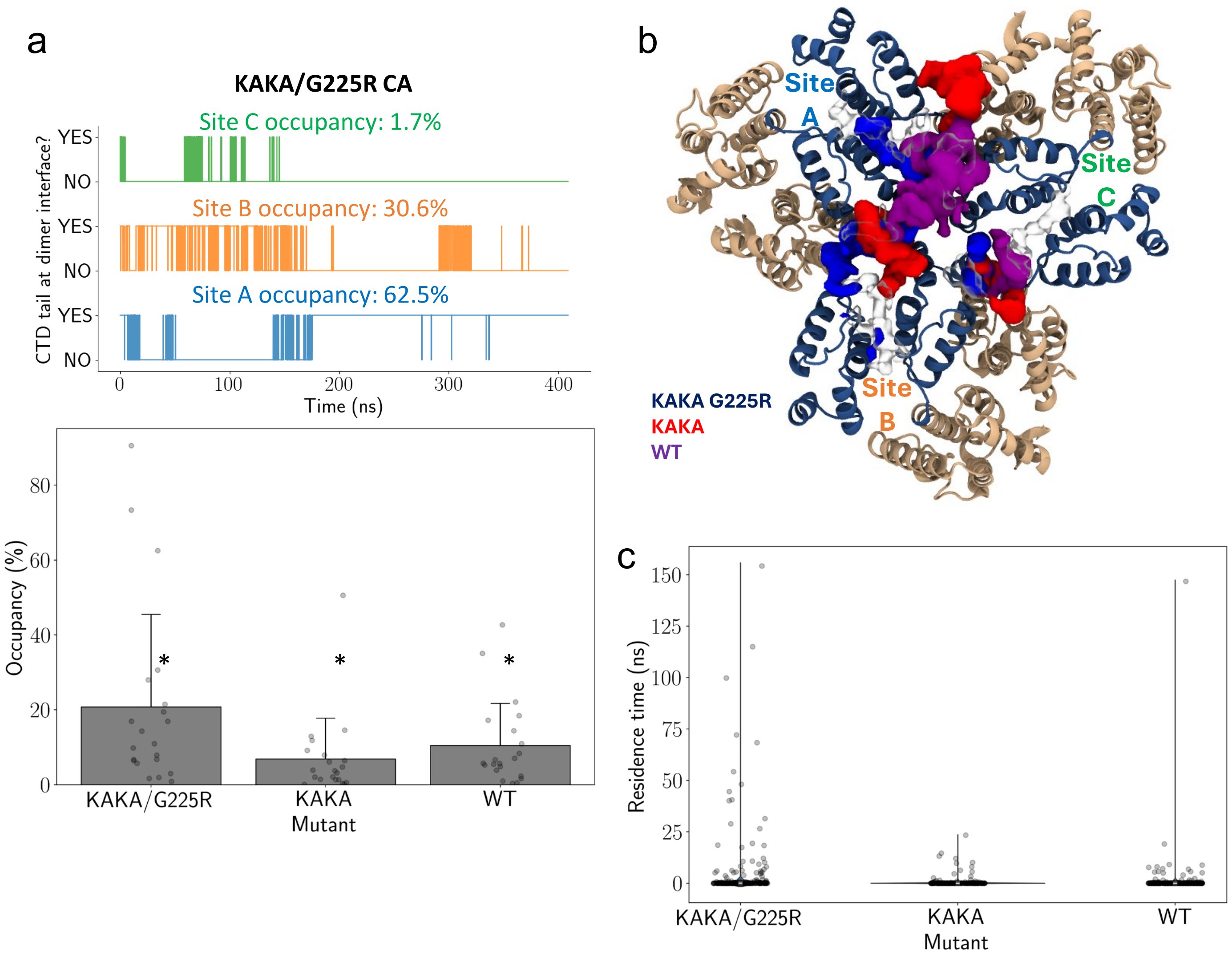

Supplementary Figure 6 | C-terminal segment occupancy from KAKA/G225R, KAKA and WT CA trimer of dimer MD simulations. (a) (Upper) Sample trace of C-terminal contacts at the CA dimer interface through a 400ns MD simulation replica for KAKA/G225R CA. The three dimer interfaces in the CA trimer of dimers are colored separately in green, orange and blue. (Lower) Average C-terminal site occupancy at the CA dimer interface across 7 400ns MD simulation replicas for KAKA/G225R CA, KAKA CA and WT CA. Error bars represent standard deviation. *n=21 measurements across all replicas. (b) Volume occupancy map derived for the position of atoms in the C-terminal segment through 400ns for KAKA/G225R CA (dark blue), KAKA CA (red) and WT CA (purple). Each dimer interface is labeled according to the colors in (a). (c) Distribution of residence times for C-terminal interactions with residues in the CA dimer interface across all CA dimer interfaces and 7 400ns MD simulation replicas for each CA sequence.

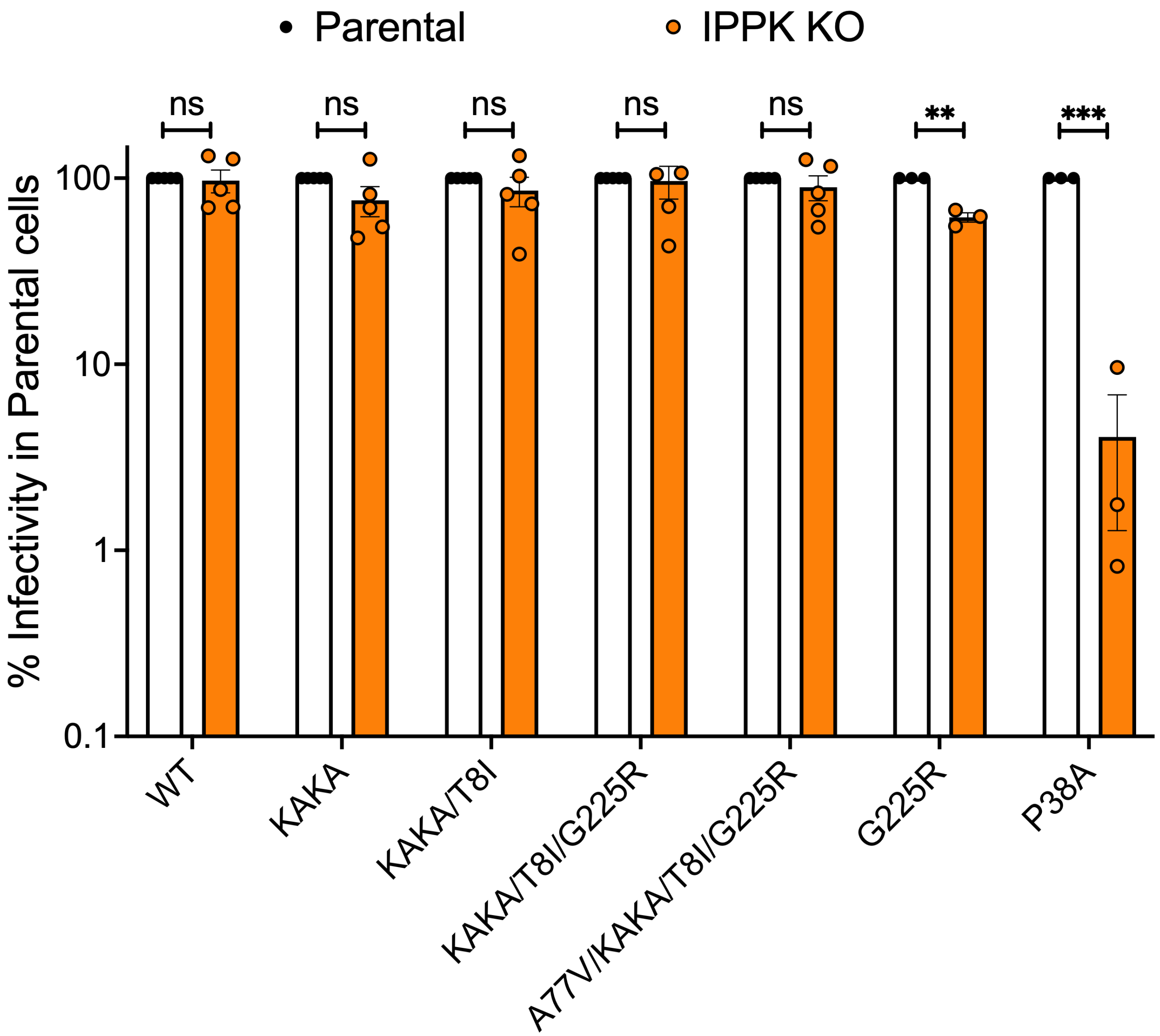

Supplementary Figure 7 | Infectivity of IP6-packaging-deficient mutants and rescue mutants is unaffected by target-cell IP6 depletion. VSV-G-pseudotyped pNL4-3.Luc.R-.E- virions were produced in HEK 293T cells and used to infect HEK 293T Parental and IPPK KO cells. Specific infectivity was measured at 48 hours post-infection and compared between each cell type for each mutant. Infectivity for each mutant in IPPK KO target cells is expressed as a percentage of that mutant's infectivity in parental target cells. Data are the mean of three independent biological replicates for G225R and P38A and five independent biological replicates for all other groups. Error bars depict +/- SEM. Statistical analysis was performed using GraphPad Prism. Statistical significance was determined by a two-tailed one-sample Student's t-test with a hypothetical value set to 100 (p-value summary: >0.05 = not significant; <0.05 = *; <0.01 = **; <0.001 = ***; <0.0001 = ****). Precise p-values for each comparison of infectivity in Parental vs. IPPK KO target cells – WT (P=8373), KAKA (P=0.1605), KAKA/T8I (0.4098), KAKA/T8I/G225R (0.8705), A77V/KAKA/T8I/G225R (0.4818), G225R (0.0083), P38A (0.0008). Source data are provided as a Source Data file.

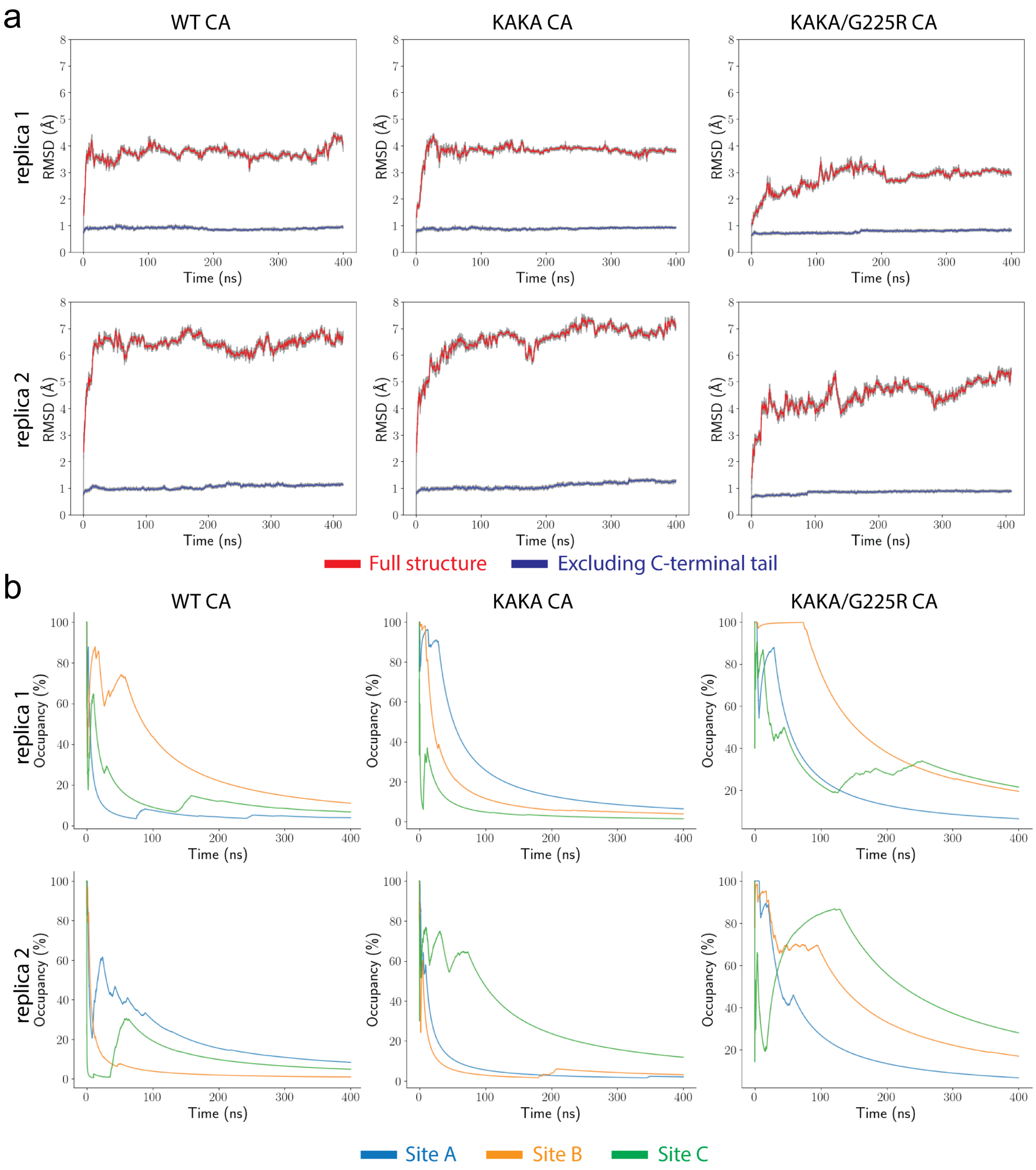

Supplementary Figure 8 | Convergence of root mean square deviation (RMSD) and C-terminal segment occupancy across MD simulations for CA trimer of dimer systems. (a) Representative RMSD plots for CA trimer of dimers for WT CA (left), KAKA CA (center) and KAKA/G225R CA (right). RMSD calculated for the entire structure (red line) and excluding the flexible C-terminal region (blue line). (b) Time-evolution of C-terminal tail occupancy at the dimer interface through the simulation for WT CA (left), KAKA CA (center) and KAKA/G225R CA (right). Each trace corresponds to occupancy at a specific CA dimer interface site, as labelled in Supplementary Figure 5. Two out of seven independent replicates are shown.
