# Supplementary material for: Structural basis for HIV-1 capsid adaption to a deficiency in IP6 packaging
Source: Nat Commun. 2025 Sep 1;16:8152. doi: 10.1038/s41467-025-63363-9 (PMC12402280; doi:10.1038/s41467-025-63363-9)
Supplement: Supplementary file 2 — Reporting Summary [file 41467_2025_63363_MOESM2_ESM.pdf]

nature portfolio

Corresponding author(s): Peijun Zhang

Last updated by author(s): Jul 27, 2025

Reporting Summary

Nature Portfolio wishes to improve the reproducibility of the work that we publish. This form provides structure for consistency and transparency in reporting. For further information on Nature Portfolio policies, see our Editorial Policies and the Editorial Policy Checklist.

Statistics

For all statistical analyses, confirm that the following items are present in the figure legend, table legend, main text, or Methods section.

n/a	Confirmed	
☐	☒	The exact sample size (*n*) for each experimental group/condition, given as a discrete number and unit of measurement
☐	☒	A statement on whether measurements were taken from distinct samples or whether the same sample was measured repeatedly
☐	☒	The statistical test(s) used AND whether they are one- or two-sided<br>*Only common tests should be described solely by name; describe more complex techniques in the Methods section.*
☒	☐	A description of all covariates tested
☒	☐	A description of any assumptions or corrections, such as tests of normality and adjustment for multiple comparisons
☐	☒	A full description of the statistical parameters including central tendency (e.g. means) or other basic estimates (e.g. regression coefficient) AND variation (e.g. standard deviation) or associated estimates of uncertainty (e.g. confidence intervals)
☐	☒	For null hypothesis testing, the test statistic (e.g. *F*, *t*, *r*) with confidence intervals, effect sizes, degrees of freedom and *P* value noted<br>*Give P values as exact values whenever suitable.*
☒	☐	For Bayesian analysis, information on the choice of priors and Markov chain Monte Carlo settings
☒	☐	For hierarchical and complex designs, identification of the appropriate level for tests and full reporting of outcomes
☒	☐	Estimates of effect sizes (e.g. Cohen's *d*, Pearson's *r*), indicating how they were calculated

Our web collection on statistics for biologists contains articles on many of the points above.

Software and code

Policy information about availability of computer code

Data collection	Tomography 5 software, EPU, SerialEM
Data analysis	MotionCor2, IMOD version 4.11, emClarity version 1.5.0.2, emClarity version 1.5.3.10, UCSF ChimeraX, cryoSPARC v4.6.0, UCSF Chimera

For manuscripts utilizing custom algorithms or software that are central to the research but not yet described in published literature, software must be made available to editors and reviewers. We strongly encourage code deposition in a community repository (e.g. GitHub). See the Nature Portfolio guidelines for submitting code & software for further information.

Data

Policy information about availability of data

All manuscripts must include a data availability statement. This statement should provide the following information, where applicable:

- Accession codes, unique identifiers, or web links for publicly available datasets
- A description of any restrictions on data availability
- For clinical datasets or third party data, please ensure that the statement adheres to our policy

All data needed to evaluate the conclusions in the paper are present in the paper and/or the Supplementary information and source data are provided with this paper. Raw data are not accessible in publicly available datasets for now due to the validation process but can be provided when requested. The immature and mature HIV-1 capsid lattice structures are deposited in the Electron Microscopy Data Bank (EMDB) and Protein Data Bank (PDB) under accession codes: EMD-51826 for the K227A immature CA hexamer, EMD-51825 for the KAKA/T8I immature CA hexamer, EMD-51821 for G225R immature CA hexamer, EMD-51822 for the

KAKA/G225R immature CA hexamer, EMD-51823 for the KAKA/T8I/G225R immature CA hexamer, EMD-51824 for the A77V/KAKA/T8I/G225R immature CA hexamer, EMD-52724 and PDB 9I8I for the KAKA/G225R mature CA hexamer, EMD-52725 for the KAKA/G225R mature CA tri-hexamer Classs 1, and EMD-52726 for the KAKA/G225R mature CA tri-hexamer Classs 2.

Research involving human participants, their data, or biological material

Policy information about studies with human participants or human data. See also policy information about sex, gender (identity/presentation), and sexual orientation and race, ethnicity and racism.

Reporting on sex and gender	n/a
Reporting on race, ethnicity, or other socially relevant groupings	n/a
Population characteristics	n/a
Recruitment	n/a
Ethics oversight	n/a

Note that full information on the approval of the study protocol must also be provided in the manuscript.

Field-specific reporting

Please select the one below that is the best fit for your research. If you are not sure, read the appropriate sections before making your selection.

☒ Life sciences ☐ Behavioural & social sciences ☐ Ecological, evolutionary & environmental sciences

For a reference copy of the document with all sections, see nature.com/documents/nr-reporting-summary-flat.pdf

Life sciences study design

All studies must disclose on these points even when the disclosure is negative.

Sample size	For HIV-1 mature cores' morphological analysis, sample sizes are listed in Figure 2h. For single particle and subtomogram averaging analysis, sample sizes are those used for resolution calculation, and are listed in Supplementary Table 1 and Supplementary Table 3.
Data exclusions	n/a
Replication	For single particle and subtomogram averaging analysis, two randomly divided half datasets were processed independently and combined to give rise to the final structures. The resolution of the structure is assessed by comparing the two independent maps
Randomization	For SPA of HIV-1 CLPs and subtomogram averaging of HIV-1 VLPs, the dataset were randomly divided into two half sets, as a standard approach implemented in cryoSPARC and Relion.
Blinding	cryo-EM and cryo-ET study including data collection and processing is not blinded.

Reporting for specific materials, systems and methods

We require information from authors about some types of materials, experimental systems and methods used in many studies. Here, indicate whether each material, system or method listed is relevant to your study. If you are not sure if a list item applies to your research, read the appropriate section before selecting a response.

Materials & experimental systems

n/a	Involved in the study	
☐	☒	Antibodies
☐	☒	Eukaryotic cell lines
☒	☐	Palaeontology and archaeology
☒	☐	Animals and other organisms
☒	☐	Clinical data
☒	☐	Dual use research of concern
☒	☐	Plants

Methods

n/a	Involved in the study	
☒	☐	ChIP-seq
☒	☐	Flow cytometry
☒	☐	MRI-based neuroimaging

Antibodies

Antibodies used	HIV-Ig (BEI resources ARP-3957); anti-human IgG HRP (Cytiva NA933)
Validation	These antibodies were validated by BEI resources and Cytiva respectively

Eukaryotic cell lines

Policy information about cell lines and Sex and Gender in Research

Cell line source(s)	MT-4, SupT1, C8166 human T, HEK 293T, TZM-bl
Authentication	Cells are authenticated by ATCC by STR profiling. MT-4, SupT1, and C8166 were authenticated after receipt by STR profiling.
Mycoplasma contamination	All the cells were tested negative for mycoplasma contamination
Commonly misidentified lines (See ICLAC register)	n/a

Plants

Seed stocks	n/a
Novel plant genotypes	n/a
Authentication	n/a
